# The relationship between prostate cancer and metabolic syndrome: A bibliometric analysis (2021–2023)

**DOI:** 10.1097/MD.0000000000049716

**Published:** 2026-07-10

**Authors:** Feng Wang, Juan Wu, Wanjiao Wang, Min Hu, Qinghong Xi

**Affiliations:** a Department of Nursing, The Ninth People’s Hospital Affiliated to Shanghai Jiao Tong University School of Medicine, Shanghai, China.

**Keywords:** bibliometric analysis, CiteSpace, metabolic syndrome, prostate cancer

## Abstract

**Background:**

Prostate cancer (PCa) is the second most common malignancy among men worldwide, and its association with metabolic syndrome (MetS) has drawn considerable attention. However, the existing literature lacks a systematic analysis of research trends and hotspots in this field.

**Methods:**

We searched for studies related to PCa and MetS from 2001 to 2023 in the Web of Science Core Collection database and then downloaded the data into CiteSpace to generate a visualization map.

**Results:**

A total of 821 articles were included, and the number of papers on the relationship between PCa and MetS showed a slow and steady growth trend over the years. The author with the highest publication volume was De Nunzio, Cosimo, and Harvard University had the most publications among the research institutions, with the United States, Italy, and China being the top 3 countries in terms of publication volume. The research hotspots mainly focused on MetS, PCa, risk, insulin resistance, men, and androgen deprivation therapy. The keyword clusters mainly included #0 benign prostatic hyperplasia, #1 testosterone, #2 cancer, #3 androgen deprivation therapy, #4 insulin resistance, #5 congenital adrenal hyperplasia, #6 disease prevention, #7 metabolic syndrome, #8 drug therapy, #9 experimental autoimmune encephalomyelitis, and #10 iron homeostasis.

**Conclusion:**

The bibliometric study results provide the current status and trends for publications in the field of PCa and MetS. MetS may be closely related to the occurrence of PCa. Early treatment of MetS is an effective approach to preventing PCa and improving prognosis. This study may help researchers identify hotspots and explore new research directions.

## 1. Introduction

The incidence rate of prostate cancer (PCa) ranks second among all male malignant tumors worldwide, accounting for 7% of newly diagnosed cancers in men globally (15% in developed regions). More than 1.2 million new cases are diagnosed every year, making it one of the main causes of male cancer-related deaths.^[1,2]^ Although PCa treatment has made recent progress, its incidence rate varies greatly among different regions of the world, yet it shows steady growth every year.^[3]^ The prognosis of individual PCa is highly variable, depending on the tumor grade and stage at initial diagnosis.^[1]^ Recognized immutable risk factors for PCa include age, race, certain genetic mutations, insulin-like growth factor, and family history, while modifiable risk factors include environmental factors, lifestyle, diet, smoking, alcohol consumption, obesity, and physical activity.^[4]^ Modifiable risk factors may affect the risk of developing PCa and dying from the disease, but apart from early diagnosis to reduce PCa mortality, there are almost no clear indications for prevention.^[5]^ In recent years, a large amount of research has focused on changing the clinical characteristics of metabolic syndrome (MetS) to prevent and treat PCa.^[6]^ MetS has become a new target for the treatment of PCa.

MetS is a chronic noninfectious syndrome characterized by a series of vascular risk factors, including insulin resistance, hypertension, obesity, impaired glucose metabolism, and lipid abnormalities. These conditions are interrelated and have underlying mediators, mechanisms, and pathways.^[7,8]^ If left untreated, MetS often progresses to more significant metabolic defects, such as type 2 diabetes and nonalcoholic fatty liver disease.^[9]^ MetS is associated with an increased risk of advanced-stage PCa and adverse pathological outcomes.^[5]^ A large clinical cohort study confirmed that increasing severity of metabolic abnormalities is also associated with an increased risk of overall and aggressive PCa diagnosis.^[10]^ To date, evidence from epidemiological studies, animal experiments, and intervention research suggests a close relationship between MetS and the diagnosis, progression, and recurrence of PCa.^[11]^ In recent years, numerous studies on the relationship between PCa and MetS have been published by scholars both domestically and internationally. However, there is a lack of a systematic review and analysis of the literature. Therefore, it is necessary to clarify the relationship between PCa and MetS, which can provide more treatment options for early intervention in PCa.

CiteSpace is a Java-based software program developed by Dr Chaomei Chen. It utilizes bibliometrics, co-occurrence analysis, and cluster analysis to analyze and visualize the hotspots and research frontiers of a specific scientific literature within a discipline or knowledge domain.^[12]^ Although the CiteSpace software has been widely used in various fields in recent years, to the best of our knowledge, there has been no bibliometric study on PCa and MetS to date. To fill this knowledge gap, this study retrieved bibliometric data related to PCa and MetS research from the Web of Science Core Collection database and conducted a visualization analysis of the current status, hotspots, and frontiers of PCa and MetS diseases using CiteSpace.

## 2. Materials and methods

### 2.1. Data collection

The Web of Science Core Collection database was selected as the source of the literature. The search period was from January 1, 2001, to December 31, 2023. The specific search formula was: “TS = (prostate neoplasm OR prostate cancer OR cancer of the prostate) AND TS = (metabolic syndrome).” The inclusion criteria were as follows: the theme was PCa and MetS; the literature types were articles and reviews; and the language was English. The exclusion criteria were as follows: literature with duplicate publication, missing information, or for which the full text could not be obtained and conference papers, newspapers, popular science articles, news reports, and academic advertisements.

### 2.2. Literature processing

**Figure 1.**
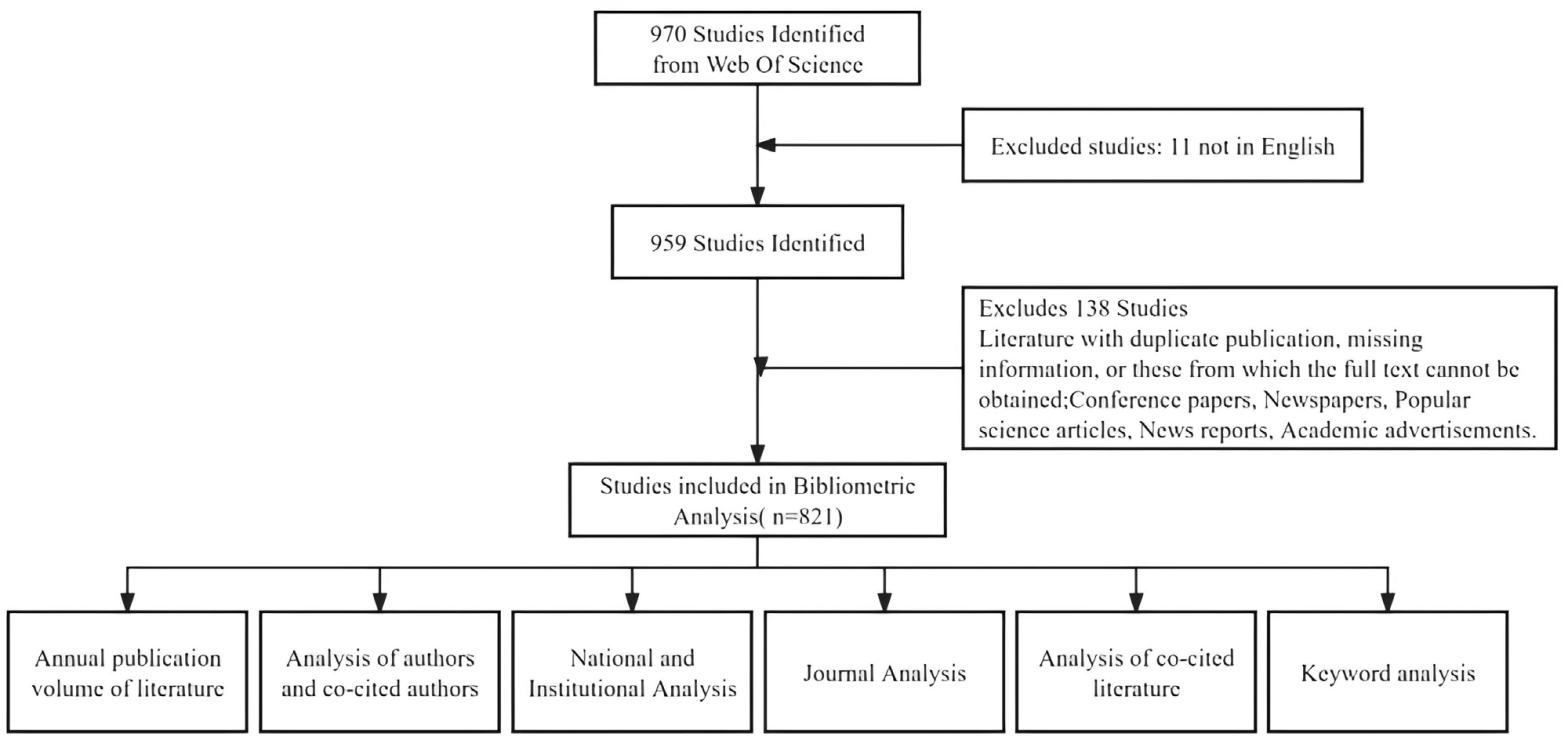
A searching flow diagram.

The retrieved literature was exported in the “Full Record and Cited References” and “Plain Text” formats, renamed as “download_*,” and then imported into the NoteExpress software (Beijing Aegean Sea Technology Co., Ltd.). Duplicate articles were removed, resulting in a final set of 821 articles. Subsequently, the articles were individually imported into CiteSpace (version 6.2.R7, Drexel University) for bibliographic visualization analysis. The time parameter was set to match the publication dates of the included literature. Different clustering maps were generated based on authors, institutions, countries, and keywords. Additionally, keyword burst analysis was conducted to generate a keyword burst map. In the network graph generated by CiteSpace, the size of the nodes and the thickness of the lines between the nodes represent the frequency of occurrence and the degree of correlation of the relevant parameters, respectively. Microsoft Excel 2021 (Microsoft Corporation) was utilized to conduct an annual publication trend analysis of the research literature and to generate charts that illustrate the global annual publication trends. The search details are presented as a flowchart in Figure 1.

### 2.3. Software parameter settings

The software parameters were set as follows: Time slice: 2001 to 2023; Slice length: 1 year; TopN%: 10%; TopN: 50; Pruning: Pathfinder and Pruning sliced networks.

## 3. Results

### 3.1. Annual publication volume of literature

**Figure 2.**
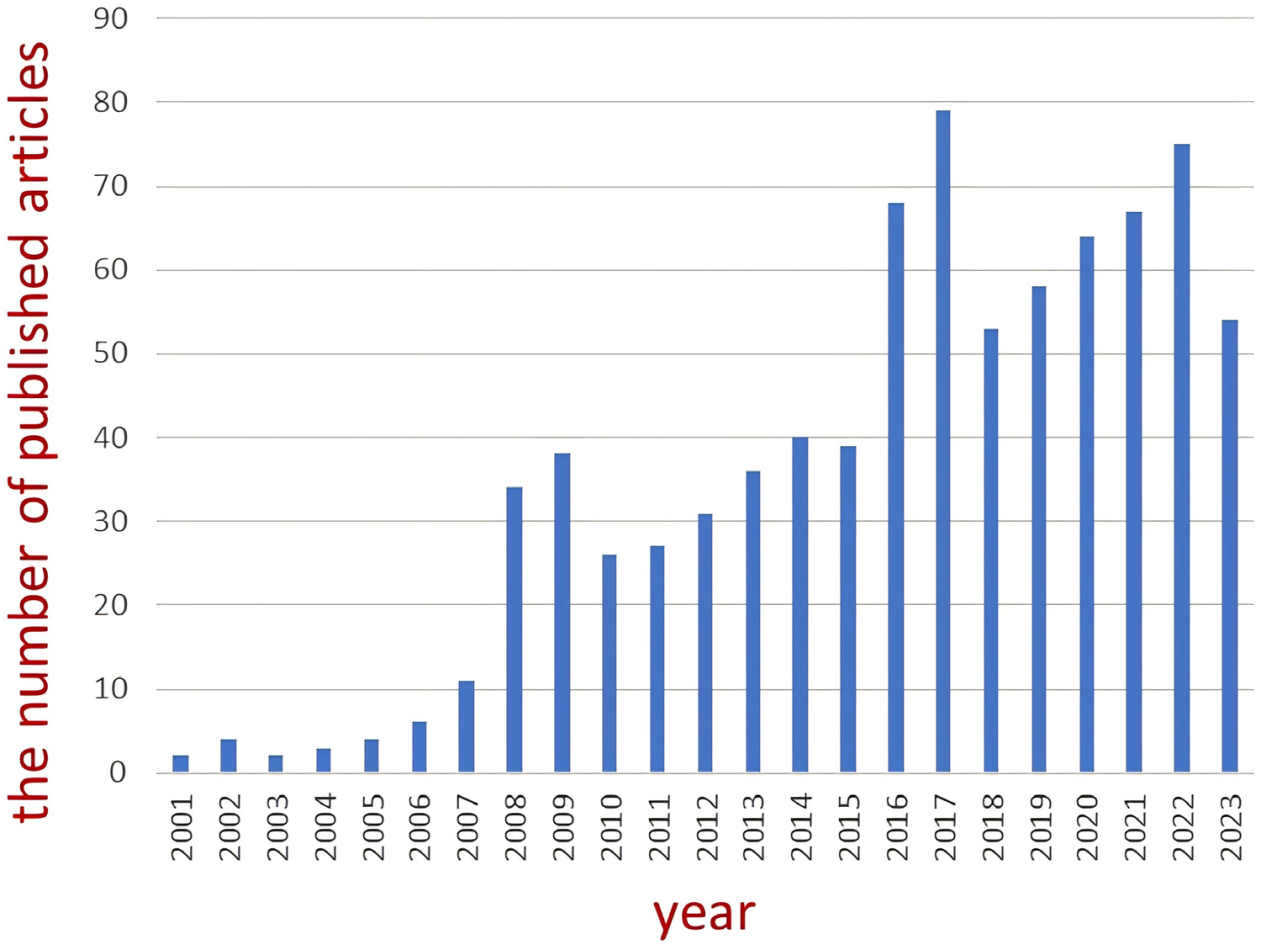
Annual publication trend of research literature on the relationship between PCa and MetS from 2001 to 2023. MetS = metabolic syndrome, PCa = prostate cancer.

This study conducted a statistical analysis of the 821 included articles based on publication time. The annual publication trend is shown in Figure 1. The total number of documents shows a fluctuating upward trend. In the early stages, the growth in publication volume was slow from 2001 to 2005; from 2007 to 2009, 2015 to 2017, and 2018 to 2022, the growth rate of publications was significant and fast, reaching 3 peaks in 2009, 2017, and 2022. There was a slight dip in the number of publications in the relevant field in 2018, yet the overall trend was upward, indicating a continued increase in research interest in the relationship between PCa and MetS, as shown in Figure 2.

### 3.2. Analysis of authors and co-cited authors

**Figure 3.**
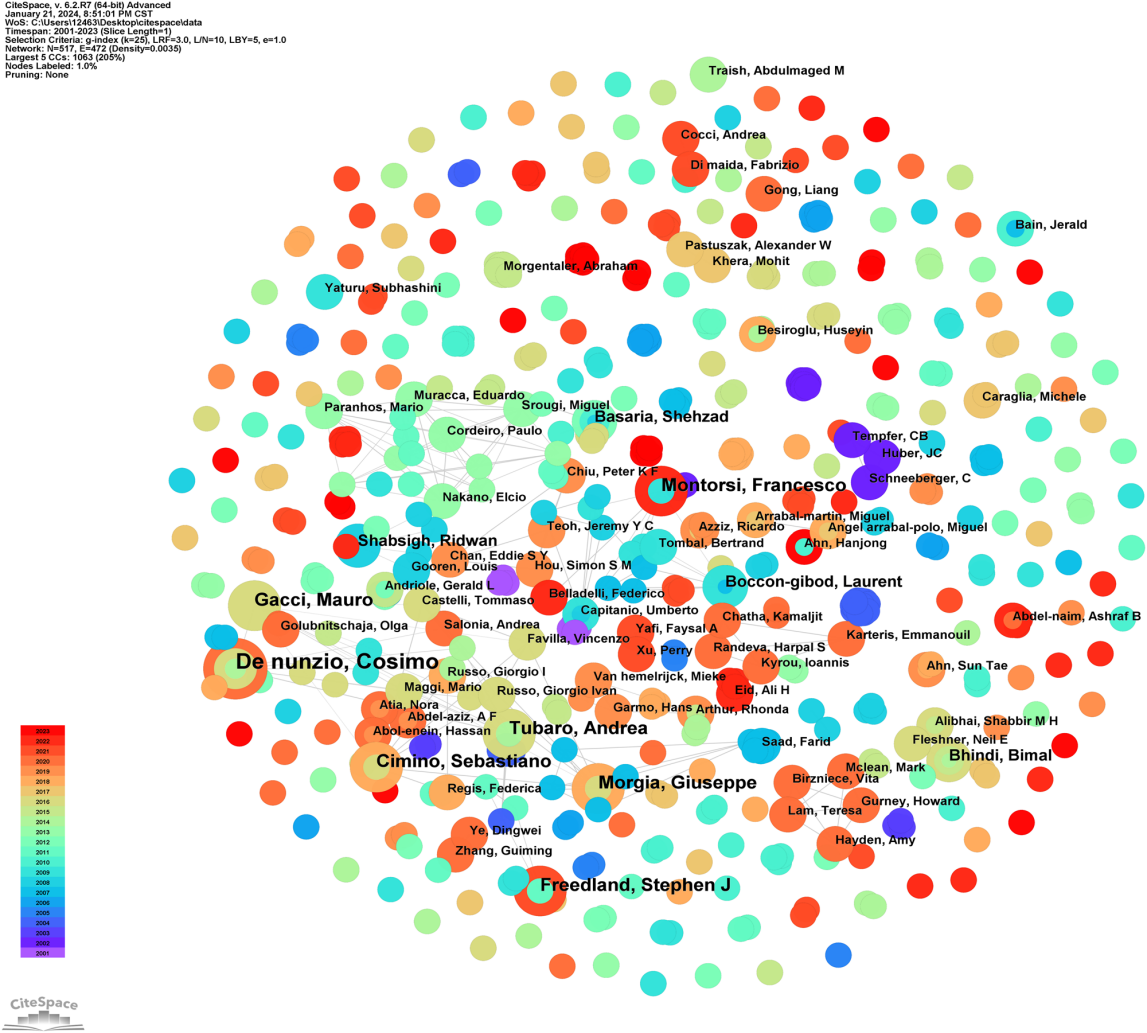
Collaborative authorship mapping of the literature on the relationship between PCa and MetS, 2001 to 2023. MetS = metabolic syndrome, PCa = prostate cancer.

A graphical analysis was performed on the distribution of authors in the publications. The results showed the number of nodes (N = 517), links (*E* = 472), and *S* = 1, representing a total of 517 authors and 472 collaborations between authors, as shown in Figure 3. Each node in the figure represents 1 author, and the larger the node, the more publications the author has. The more connections between authors, the higher the degree of closeness between them. Table 1 shows the top 10 most active authors, with De Nunzio, Cosimo, from Italy ranking first with 6 publications. In 2014, De Nunzio, Cosimo’s team conducted a prospective single-cohort study and found that patients with MetS had a higher risk of developing PCa.^[13]^ The team launched a multicenter cohort study on radical prostatectomy in 2018 to explore the relationship between MetS and PCa.^[14]^ The next authors on the list are Gacci, Mauro (n = 4), Morgia, Giuseppe (n = 4), Freedland, Stephen J (n = 4), Montorsi, Francesco (n = 4), Tubaro, Andrea (n = 4), and Cimino, Sebastiano (n = 4). Multiple team clusters have been formed among authors, mainly led by 3 research teams represented by De Nunzio, Cosimo; Montorsi, Francesco; and Cordeiro, respectively. The members within each team collaborate more closely, but there is a lack of cooperation between different teams, with little academic exchange between most research teams, and some researchers have not yet formed research teams. The centrality of each author is 0.00 (<0.1), which means that there is still a lack of influential and prestigious research authors at present.

**Table 1:** Top 10 authors with published papers and citations on the relationship between PCa and MetS.

Rank	Author	Frequency	Centrality	Rank	Cited author	Frequency	Centrality
1	De Nunzio, Cosimo	6	0.00	1	Anonymous	177	0.00
2	Gacci, Mauro	4	0.00	2	Smith MR	99	0.00
3	Morgia, Giuseppe	4	0.00	3	Basaria S	92	0.00
4	Freedland, Stephen J	4	0.00	4	De Nunzio C	78	0.00
5	Montorsi, Francesco	4	0.00	5	Keating NL	77	0.00
6	Tubaro, Andrea	4	0.00	6	Giovannucci E	76	0.00
7	Cimino, Sebastiano	4	0.00	7	Corona G	75	0.00
8	Bhindi, Bimal	3	0.00	8	Braga-Basaria M	71	0.00
9	Basaria, Shehzad	3	0.00	9	Bhasin S	71	0.00
10	Shabsigh, Ridwan	3	0.00	10	Grundy SM	68	0.00

MetS = metabolic syndrome, PCa = prostate cancer.

**Figure 4.**
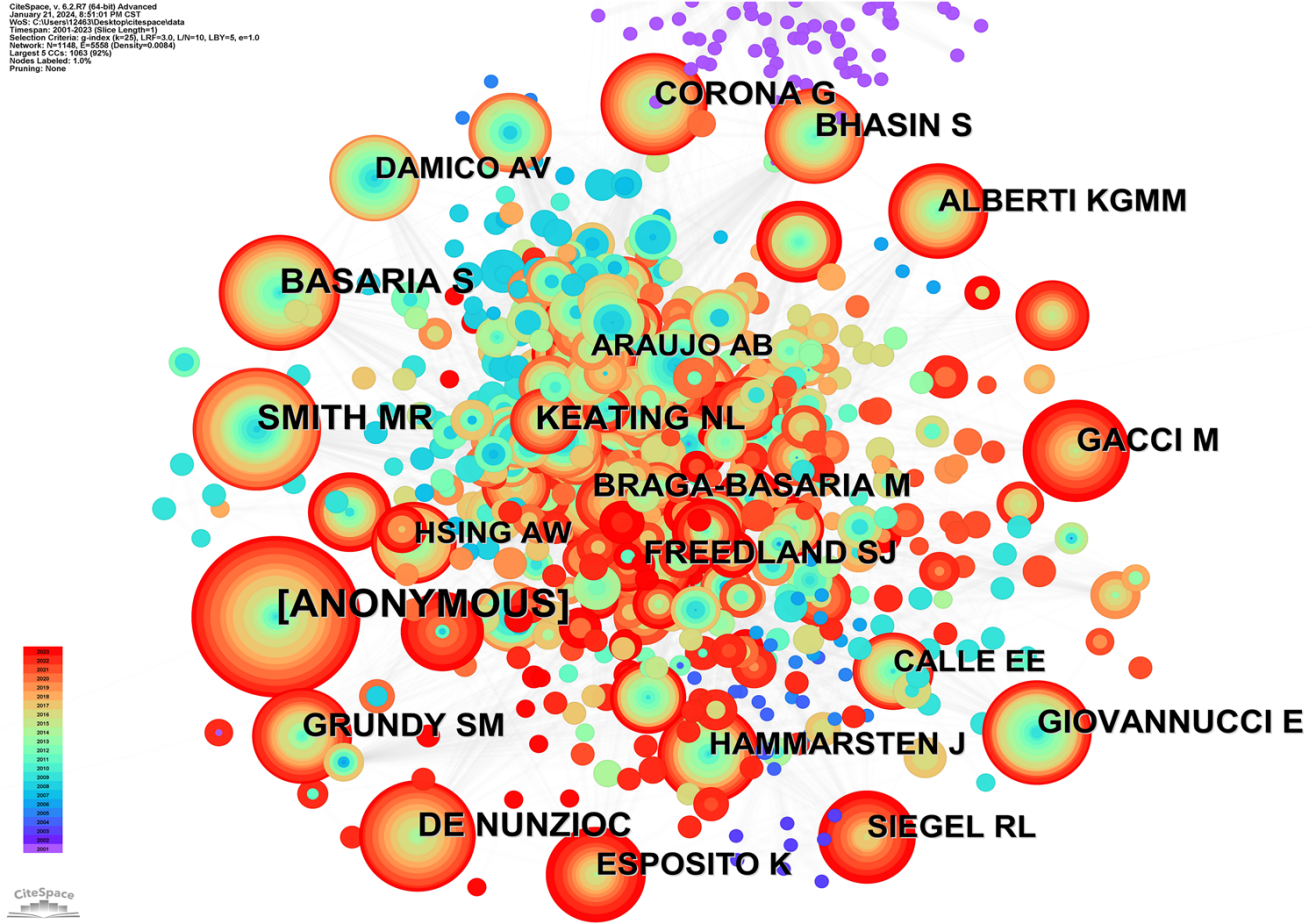
The author co-citation map.

Select “Cited Author” in the CiteSpace node types, where the size of the node represents the frequency with which the author is cited. In the obtained graph, there are a total of 1148 authors and 5558 author-to-author connections, as shown in Figure 4. The author with the highest citation frequency is Anonymous (177), followed by Smith MR (99), Basaria S (92), and De Nunzio C (78). The top 10 cited authors in this field are shown in Table 1.

### 3.3. National and institutional analysis

**Figure 5.**
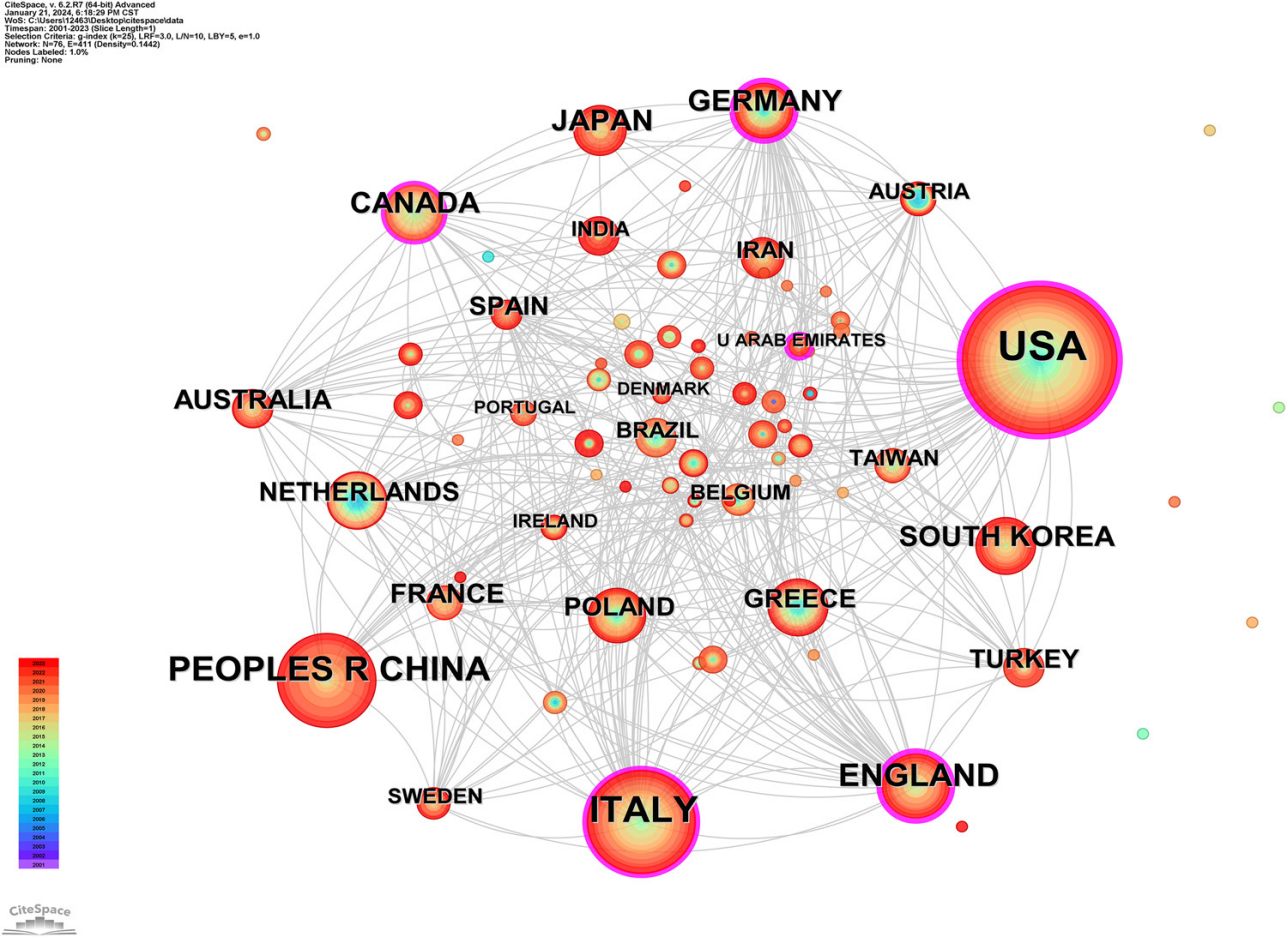
National collaboration map of research literature on the relationship between PCa and MetS. MetS = metabolic syndrome, PCa = prostate cancer.

From 2001 to 2023, 821 published papers on the relationship between PCa and MetS were contributed by 76 countries (Fig. 5). Each node represents a country, and each link represents a collaboration. There are 411 collaboration ties between countries, with a network density of 0.1442. Among these countries, the top 10 accounted for 728 studies, which is 88.7% of all publications. In terms of publication quantity, as shown in Table 2, the country with the most publications was the United States (n = 232), followed by Italy (n = 120), China (n = 86), and the United Kingdom (n = 57). The country with the highest centrality was the United States (0.33), followed by Germany (0.22), indicating that the United States and Germany played important roles in international collaborations between countries.

**Table 2:** Top 10 countries and institutions in terms of the number of published studies from 2001 to 2023.

Rank	Country	Frequency	Centrality	Rank	Institution	Frequency	Centrality
1	United States	232	0.33	1	Harvard University	25	0.13
2	Italy	120	0.13	2	University of Florence	21	0.04
3	People’s Republic of China	86	0.07	3	Sapienza University Rome	19	0.07
4	United Kingdom	57	0.21	4	University of Toronto	17	0.00
5	Canada	46	0.11	5	Azienda Ospedaliera Sant’Andrea	14	0.02
6	Germany	43	0.22	6	University of Catania	12	0.01
7	Japan	42	0	7	Azienda Ospedaliero Universitaria Careggi	12	0.02
8	South Korea	39	0.03	8	Boston University	12	0.03
9	Australia	32	0.07	9	Vita-Salute San Raffaele University	12	0.06
10	France	31	0.05	10	Harvard Medical School	11	0.02

**Figure 6.**
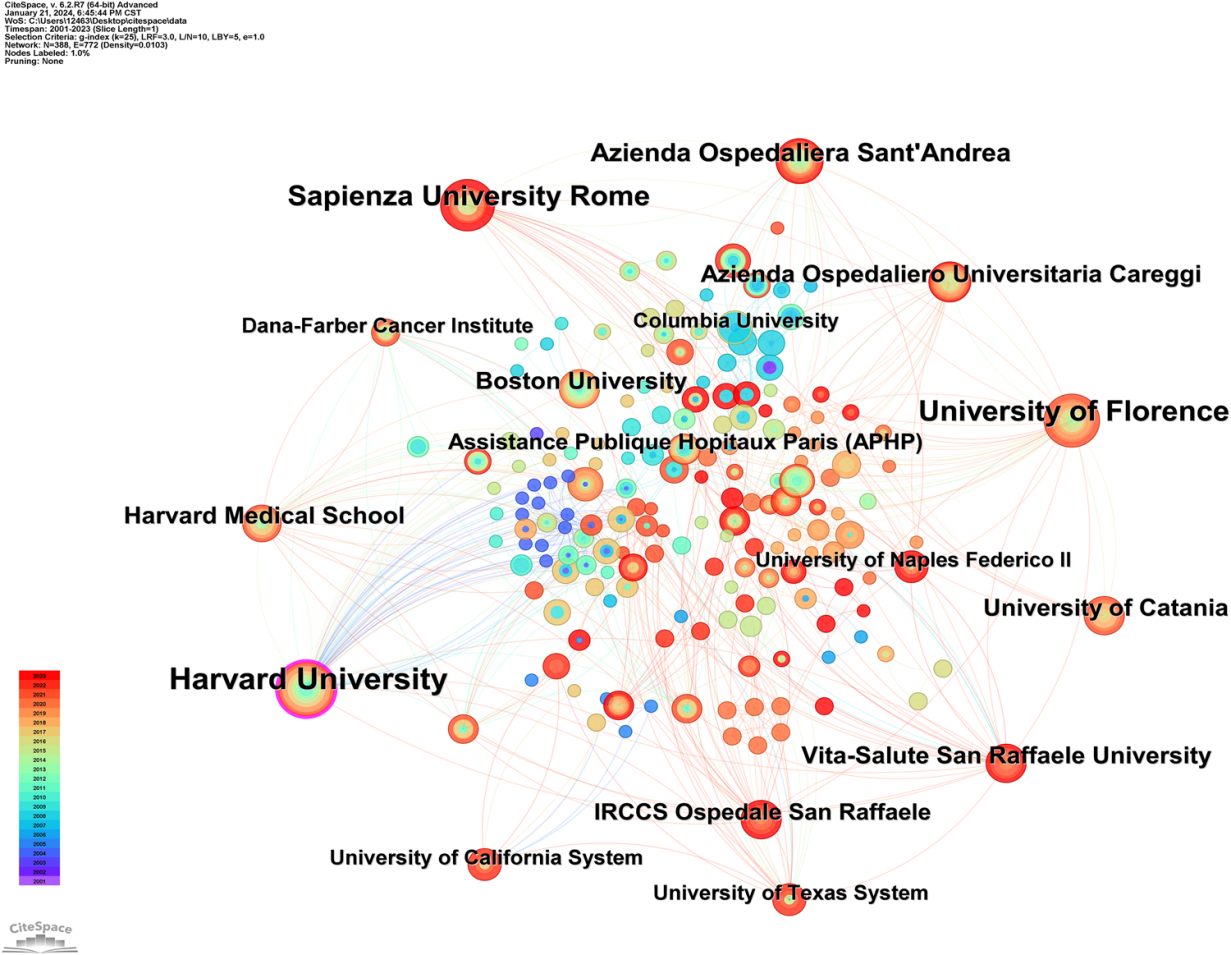
Institutional collaboration map of literature on the relationship between PCa and MetS. MetS = metabolic syndrome, PCa = prostate cancer.

Figure 6 illustrates the achievements and collaboration among research institutions, with a total of 388 nodes forming 772 connections. The size of each node represents the publication output of the corresponding research institution, while the width and number of connections indicate the level of collaboration and closeness between institutions. From 2001 to 2013, a total of 388 research institutions published articles related to PCa and MetS. Harvard University ranked first in terms of publication output with a total of 25 articles, followed by the University of Florence (21), Sapienza University Rome (19), and the University of Toronto (17). Research strength was concentrated in higher education institutions, with the top 3 institutions publishing 65 articles, accounting for 7.91% of all publications.

### 3.4. Journal analysis

**Figure 7.**
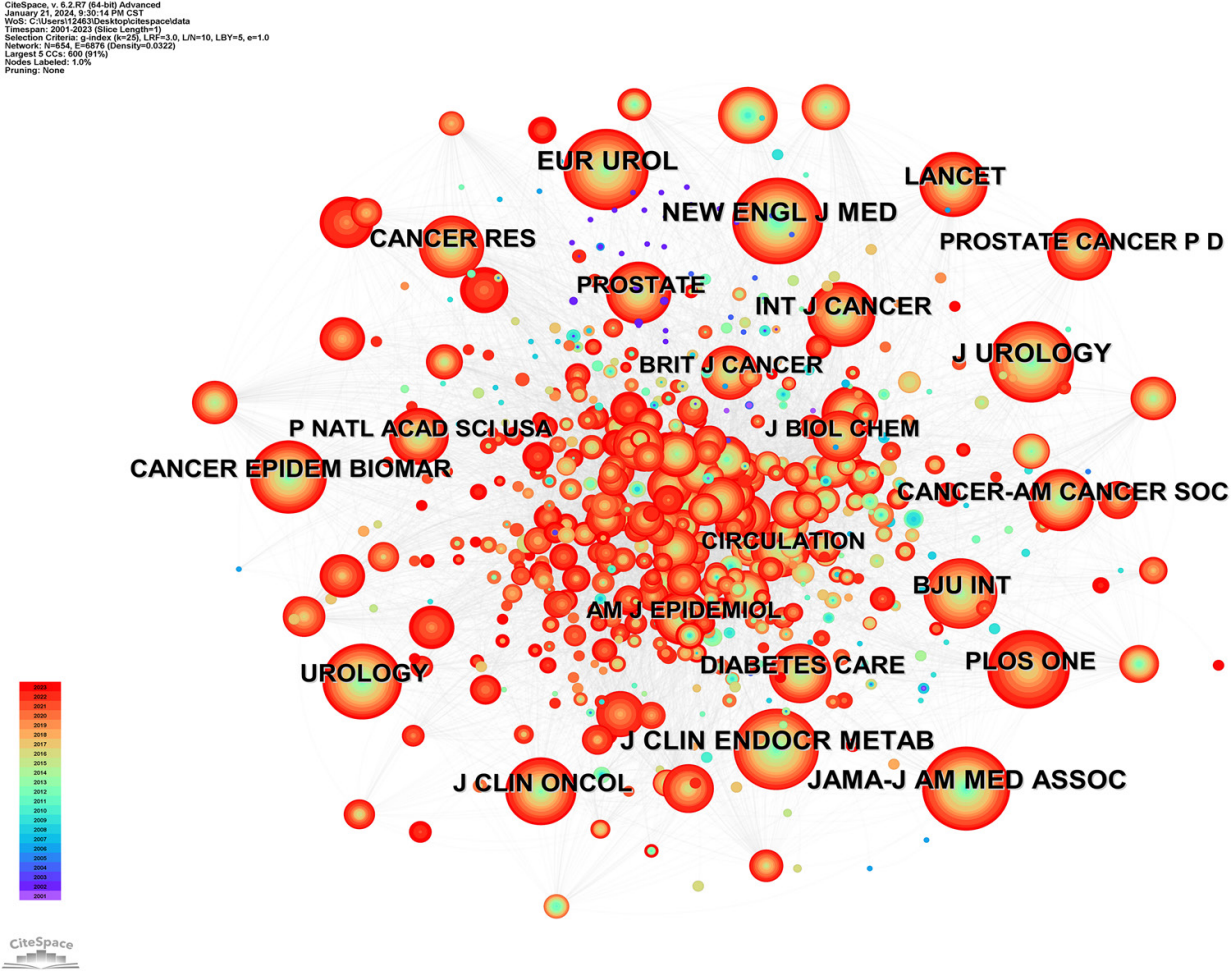
Co-cited journals on the relationship between PCa and MetS. MetS = metabolic syndrome, PCa = prostate cancer.

The highest-ranked journal based on citation count was New Engl J Med, with 399 citations, followed by J Urology (379 citations), J Clin Endocr Metab (378 citations), Eur Urol (370 citations), and JAMA-J Am Med Assoc (359 citations; Fig. 7). Among them, the top 10 journals in terms of citation centrality and citation frequency are shown in Table 3. The impact factors of the top 3 journals with the highest citation counts were all >5, with the highest impact factor reaching 158.5. This clearly demonstrates the relatively high status of the research field examining the relationship between PCa and MetS and indicates that it is receiving considerable attention from researchers.

**Table 3:** The top 10 journals in terms of citation frequency and centrality in the relationship between PCa and MetS.

Rank	Frequency	Cited journal	Impact factor	Centrality	Cited journal	Impact factor
1	399	New Engl J Med	158.5	0.07	Biochem Bioph Res Co	3.1
2	379	J Urology	6.6	0.05	Cell	64.5
3	378	J Clin Endocr Metab	5.8	0.05	Cancer Lett	9.7
4	370	Eur Urol	23.4	0.05	Carcinogenesis	4.7
5	359	JAMA-J Am Med Assoc	1.6	0.04	Cancer Res	11.2
6	340	Urology	2.1	0.04	J Biol Chem	4.8
7	322	BJU Int	4.5	0.04	Endocrinology	4.8
8	313	PLoS One	3.7	0.04	J Clin Invest	15.9
9	290	Cancer Epidem Biomar	3.8	0.04	Diabetes	7.7
10	289	J Clin Oncol	45.3	0.04	Endocr Rev	20.3

MetS = metabolic syndrome, PCa = prostate cancer.

### 3.5. Analysis of co-cited literature

The highly co-cited literature in the field of research on the relationship between PCa and MetS demonstrates the research hotspots, mainstream perspectives, and directions in this field. The top 10 co-cited papers are listed in Table 4. The most co-cited paper is “Diabetes and Cardiovascular Disease during Androgen Deprivation Therapy for Prostate Cancer” (Keating NL, 2006), cited 28 times. This study conducted an observational study on a population of 73,196 individuals aged 66 years or older and concluded that the use of gonadotropin-releasing hormone (GnRH) agonists for localized PCa in men may be associated with an increased risk of diabetes and cardiovascular disease. The benefits of GnRH agonist therapy should be weighed against these potential risks. More research is needed to identify the population at highest risk for treatment-related complications and to develop strategies for the prevention and management of treatment-related diabetes and cardiovascular disease.^[15]^ The next highly cited paper is “Meta-analysis of metabolic syndrome and prostate cancer” (Gacci M, 2017). This study is a meta-analysis that included 24 studies involving a total of 132,589 subjects. The study found that the presence of MetS is associated with poor oncological outcomes in PCa patients, particularly more aggressive tumor characteristics and biochemical recurrence.^[16]^

**Table 4:** List of co-cited literature ranked in the top 10 by frequency.

Rank	Frequency	Centrality	Year	Author	Title	Journal
1	28	0	2006	Keating NL	Diabetes and cardiovascular disease during androgen deprivation therapy for prostate cancer	J Clin Oncol
2	26	0	2017	Gacci M	Meta-analysis of metabolic syndrome and prostate cancer	Prostate Cancer P D
3	25	0	2007	Tsai HK	Androgen deprivation therapy for localized prostate cancer and the risk of cardiovascular mortality	J Natl Cancer I
4	25	0	2017	Siegel RL	Cancer statistics, 2017	CA-Cancer J Clin
5	24	0	2006	Smith MR	Insulin sensitivity during combined androgen blockade for prostate cancer	J Clin Endocr Metab
6	23	0	2012	De Nunzio C	The correlation between metabolic syndrome and prostatic diseases	Eur Urol
7	22	0	2006	Braga-Basaria M	Metabolic syndrome in men with prostate cancer undergoing long-term androgen-deprivation therapy	J Clin Oncol
8	21	0	2007	Saigal CS	Androgen deprivation therapy increases cardiovascular morbidity in men with prostate cancer	Cancer-Am Cancer Soc
9	20	0	2007	D’Amico AV	Influence of androgen suppression therapy for prostate cancer on the frequency and timing of fatal myocardial infarctions	J Clin Oncol
10	18	0	2010	Basaria S	Adverse events associated with testosterone administration	New Engl J Med

### 3.6. Keyword analysis

#### 3.6.1. Keyword co-occurrence

**Figure 8.**
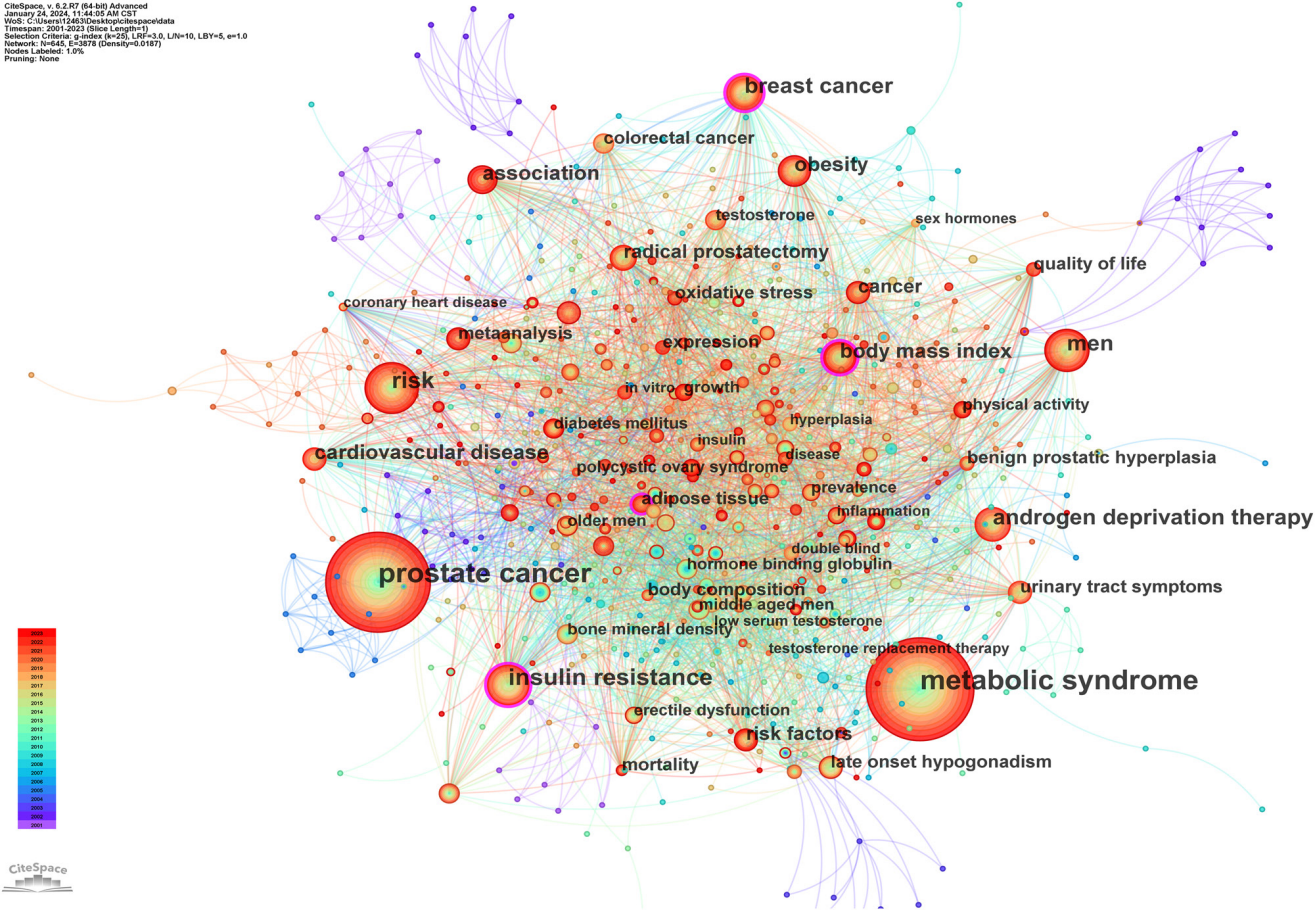
Keyword co-occurrence of literature on the relationship between PCa and MetS. MetS = metabolic syndrome, PCa = prostate cancer.

Keyword co-occurrence can reflect the hot topics in a specific field. In the co-occurrence network graph, nodes represent corresponding keywords, and the size of the nodes represents the number of articles containing the keywords. The connections between nodes represent the relationships between keywords. High-centrality keywords indicate the status and importance of the corresponding research topics in the field, while high-frequency keywords reflect popular themes.^[17]^ In this study, a keyword co-occurrence graph related to the relationship between PCa and MetS was generated using CiteSpace, as shown in Figure 8. The top 10 high-frequency keywords are MetS, prostate cancer, risk, insulin resistance, men, androgen deprivation therapy, breast cancer, body mass index, obesity, and association. The top 10 high-centrality keywords are body mass index, breast cancer, insulin resistance, adipose tissue, benign prostatic hyperplasia, cancer, androgen deprivation therapy, bone mineral density, prostate cancer, and colorectal cancer. This information is presented in Table 5.

**Table 5:** Top 10 keyword frequency and centrality ranking in the study of the relationship between PCa and MetS.

Rank	Frequency	Keywords	Rank	Centrality	Keywords
1	462	metabolic syndrome	1	0.16	body mass index
2	438	prostate cancer	2	0.11	breast cancer
3	139	risk	3	0.11	insulin resistance
4	132	insulin resistance	4	0.11	adipose tissue
5	122	men	5	0.08	benign prostatic hyperplasia
6	103	androgen deprivation therapy	6	0.08	cancer
7	94	breast cancer	7	0.08	androgen deprivation therapy
8	87	body mass index	8	0.07	bone mineral density
9	78	obesity	9	0.07	prostate cancer
10	75	association	10	0.07	colorectal cancer

MetS = metabolic syndrome, PCa = prostate cancer.

#### 3.6.2. Keyword clustering

**Figure 9.**
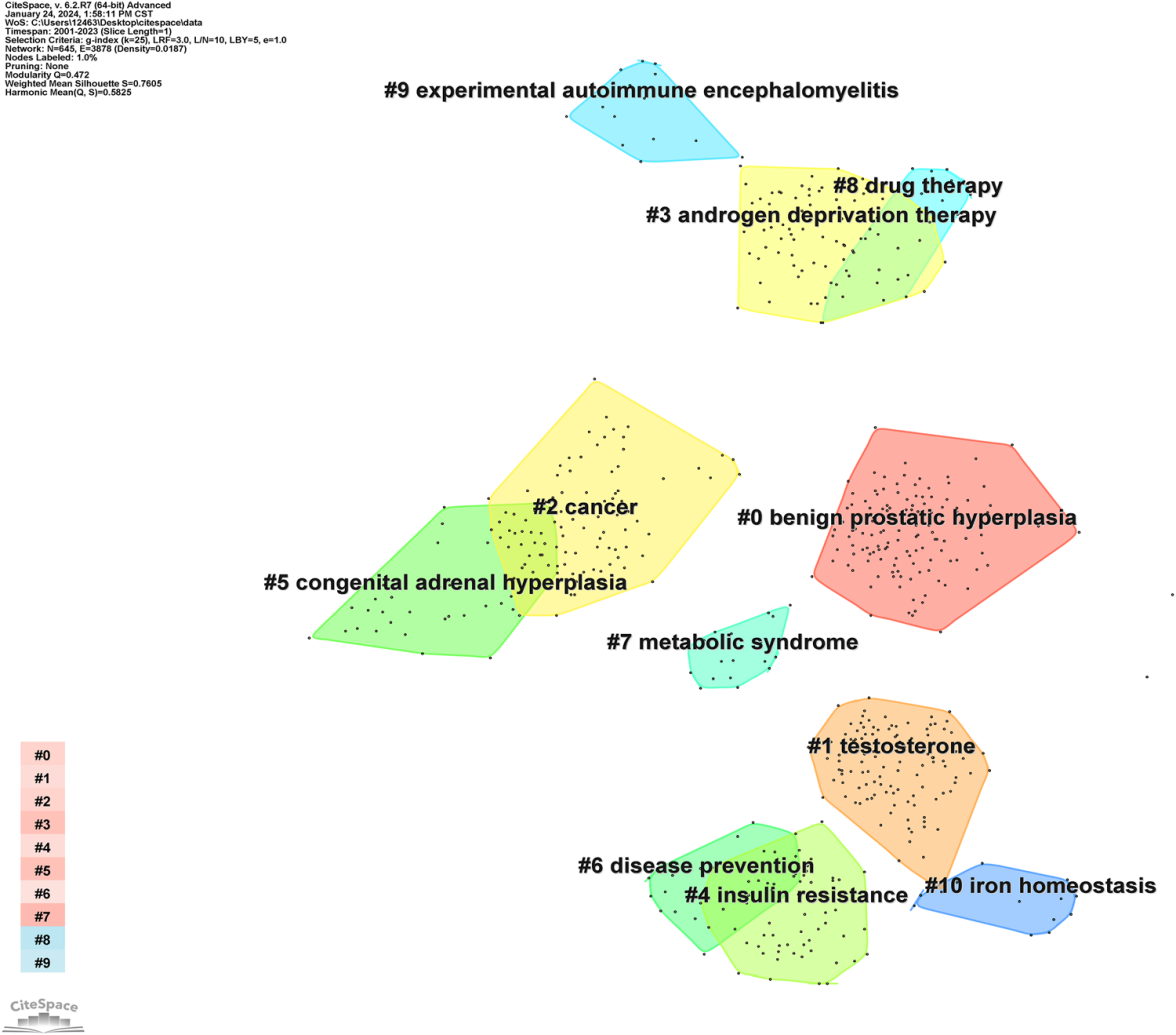
Keyword clustering of literature on the relationship between PCa and MetS. MetS = metabolic syndrome, PCa = prostate cancer.

CiteSpace was used to conduct cluster analysis on keywords, employing the log-likelihood ratio algorithm. By extracting keywords from the literature, cluster naming was achieved, and the cluster labels revealed the main themes in the research field. In the end, 11 clusters were obtained, with silhouette values above 0.6, indicating that the clustering results were reliable and meaningful^[18]^ (Table 6). The modularity *Q* = 0.472 (>0.3), indicating a significant clustering structure that could clearly define the research directions of each cluster. The mean silhouette = 0.7605 (>0.7), indicating good homogeneity and high reliability among the clusters. The extracted cluster label keywords are as follows: #0 benign prostatic hyperplasia, #1 testosterone, #2 cancer, #3 androgen deprivation therapy, #4 insulin resistance, #5 congenital adrenal hyperplasia, #6 disease prevention, #7 metabolic syndrome, #8 drug therapy, #9 experimental autoimmune encephalomyelitis, and #10 iron homeostasis (Fig. 9).

**Table 6:** Keyword clustering labels for the relationship between PCa and MetS.

Cluster-ID	Size	Silhouette	Keyword (top 5)	Label
0	128	0.618	benign prostatic hyperplasia (34.66, 1.0E−4); prostate-specific antigen (21.26, 1.0E−4); luts (17.14, 1.0E−4); lower urinary tract symptoms (16.49, 1.0E−4); men (13.76, 0.001)	Benign prostatic hyperplasia
1	116	0.767	testosterone (55.76, 1.0E−4); hypogonadism (50.91, 1.0E−4); androgen deprivation therapy (40.5, 1.0E−4); testosterone replacement therapy (25.7, 1.0E−4); erectile dysfunction (18.26, 1.0E−4)	Testosterone
2	91	0.711	cancer (14.46, 0.001); colorectal cancer (13.96, 0.001); pancreatic cancer (13.96, 0.001); testosterone (12.68, 0.001); ghrelin (12.28, 0.001)	Cancer
3	81	0.686	testosterone (14.4, 0.001); androgen deprivation therapy (11.02, 0.001); biochemical recurrence (9.64, 0.005); polyphenols (9.64, 0.005); hypogonadism (9.33, 0.005)	Androgen deprivation therapy
4	61	0.737	insulin resistance (30.98, 1.0E−4); polycystic ovary syndrome (28.39, 1.0E−4); lung cancer (12.59, 0.001); coronary heart disease (10.83, 0.001); growth factor i (10.81, 0.005)	Insulin resistance
5	37	0.871	congenital adrenal hyperplasia (cah) (13.4, 0.001); castration resistant prostate cancer (crpc) (13.4, 0.001); polycystic ovary syndrome (pcos) (13.4, 0.001); depression (6.88, 0.01); immunoassay (6.69, 0.01)	Congenital adrenal hyperplasia
6	18	0.97	disease prevention (17.06, 1.0E−4); type 2 diabetes (8.51, 0.005); alpha linolenic acid (8.51, 0.005); dietary flaxseed (8.51, 0.005); cohort study (8.51, 0.005)	Disease prevention
7	18	0.969	metabolic syndrome (15.9, 1.0E−4); aggressive metastatic disease (11.05, 0.001); 3pm (11.05, 0.001); multi-omics (11.05, 0.001); risk assessment (11.05, 0.001)	Metabolic syndrome
8	17	0.98	drug therapy (30.75, 1.0E−4); highly active (10.15, 0.005); sub-saharan africa (10.15, 0.005); cardiomyopathy (10.15, 0.005); hiv infections (10.15, 0.005)	Drug therapy
9	15	0.983	experimental autoimmune encephalomyelitis (9.79, 0.005); lynch syndrome (9.79, 0.005); vitamin d supplementation (9.79, 0.005); mismatch repair system (9.79, 0.005); helicobacter pylori infection (9.79, 0.005)	Experimental autoimmune encephalomyelitis
10	12	0.984	iron homeostasis (12.16, 0.001); thalidomide (12.16, 0.001); therapeutic use (12.16, 0.001); metalloreductase (12.16, 0.001); pharmacology (9.39, 0.005)	Iron homeostasis

MetS = metabolic syndrome, PCa = prostate cancer.

#### 3.6.3. Keyword time zone map

**Figure 10.**
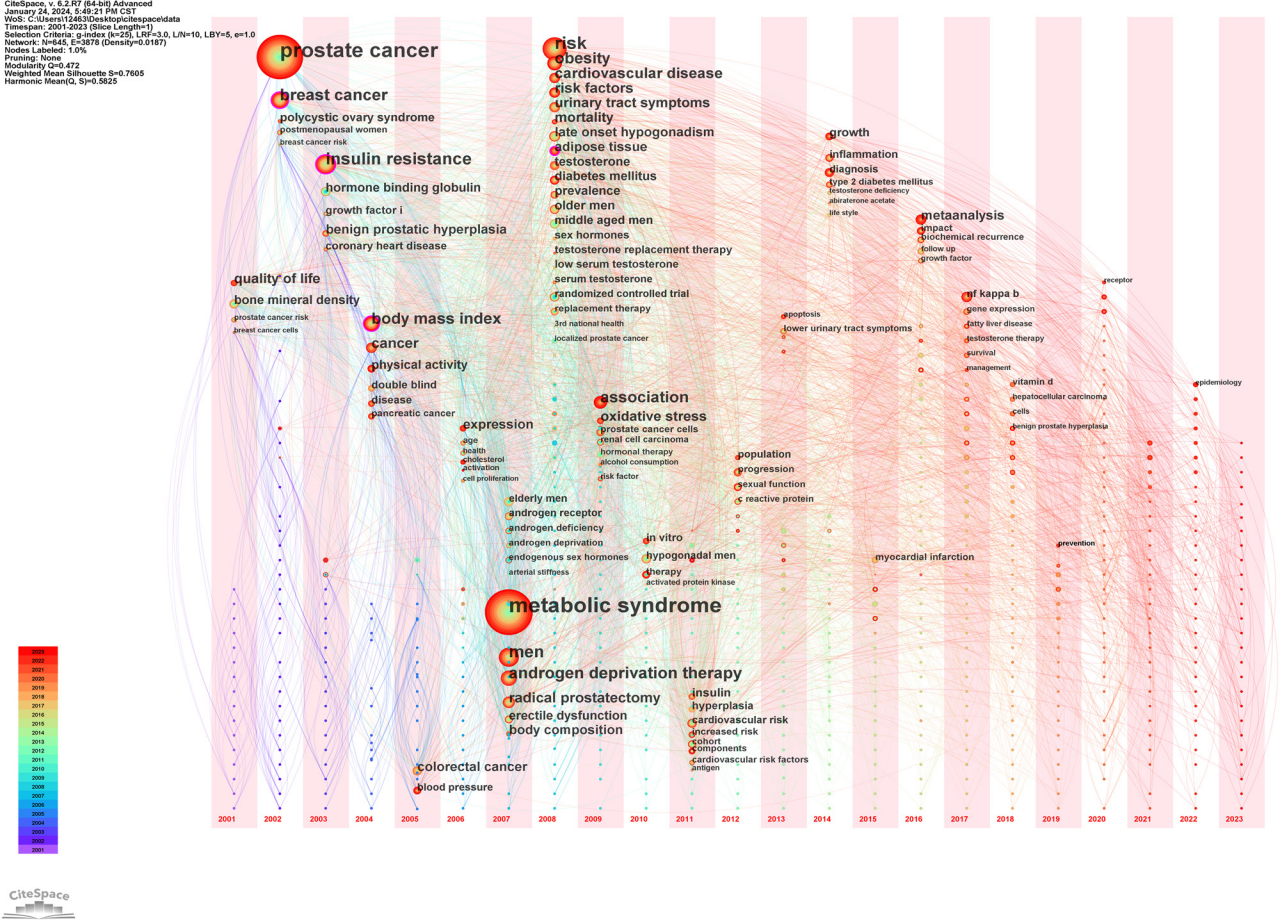
Keyword time zone map for the study of the relationship between PCa and MetS. MetS = metabolic syndrome, PCa = prostate cancer.

The keyword time zone map presents the evolving trends and mutual influences of the research frontier in the field over time in the form of a time series graph. This map can demonstrate the overall development trajectory of the research field, as shown in Figure 10. In the graph, keywords are arranged from left to right based on their first appearance over time, with the size of the nodes and font indicating the frequency of keyword occurrence. In 2001, the main keywords were quality of life, bone mineral density, and PCa risk, while in recent years, keywords such as epidemiology have dominated. Research on the relationship between PCa and MetS became more prevalent in 2009, indicating that the correlation between PCa and MetS has attracted attention from experts in this field.

#### 3.6.4. Keyword burst detection

**Figure 11.**
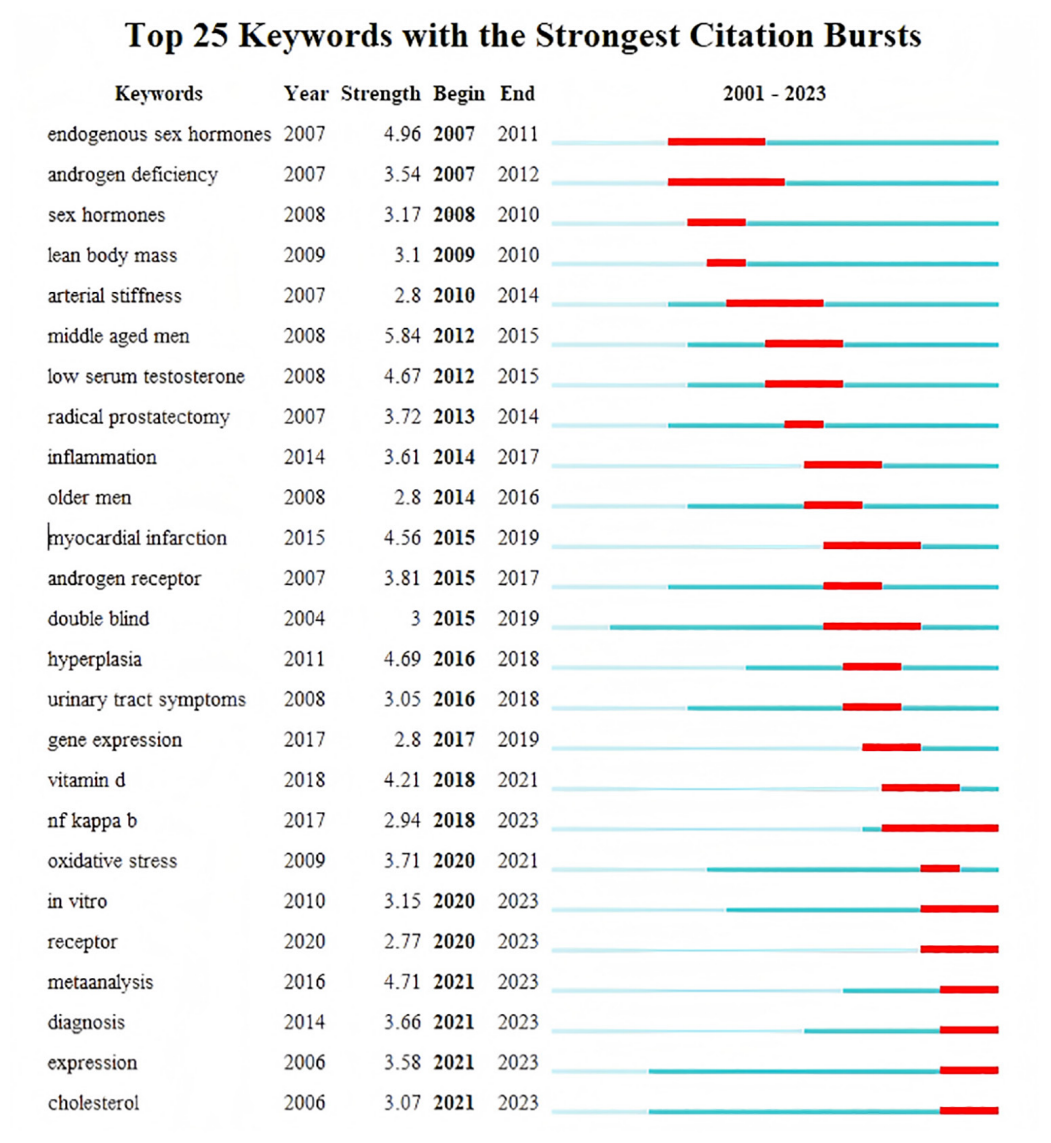
Top 25 keywords with citation burst.

Burst keywords refer to the increased frequency of certain keywords within a certain period, representing research hotspots and trends during that period. “Begin” represents the starting time of the burst, “end” represents the ending time, and “strength” represents the intensity of the keyword mutation, with higher strength indicating greater influence. In the field of research on the relationship between PCa and MetS, a total of 25 burst keywords were detected in the burst keyword map (Fig. 11), among which “endogenous sex hormones,” “androgen deficiency,” and “sex hormones” were the earliest emerging words, while “androgen deficiency” and “NF-kappa B” had the longest duration as burst keywords.

## 4. Discussion

PCa is one of the most common malignant tumors and a leading cause of cancer-related deaths worldwide.^[19]^ It typically occurs in individuals aged 50 and above, originating from abnormal cells in the prostate tissue. If left untreated, it can spread to nearby tissues, lymph nodes, and even the bones.^[20]^ Research has demonstrated the presence of complex interactions between MetS and the risk of PCa, as treatment for one condition may affect the other.^[6]^ Therefore, a bibliometric analysis of the relationship between PCa and MetS is necessary.

This study found that research on the correlation between PCa and MetS showed a rapid development trend between 2010 and 2017, which may be closely related to the increasing incidence of PCa worldwide in recent years.^[21]^ Among the research countries and institutions in this field, Harvard University in the United States ranked first in terms of publication quantity and collaboration intensity. China ranked third in terms of publication quantity, but its centrality value was only 0.07, indicating limited communication and collaboration among institutions. This suggests that Chinese researchers should pay more attention to the breadth and depth of research on the correlation between PCa and MetS, and there is a need for increased collaboration with foreign research teams.

The analysis of the co-citation literature network can help identify the most valuable literature in the field of PCa and MetS. Among the top 10 co-cited articles identified in this study, 6 focused on the impact of androgen deprivation therapy on the relationship between PCa and MetS.^[15,22–26]^ GnRH agonist androgen deprivation therapy is the cornerstone of treatment for metastatic PCa and a routine management approach for many men with localized and locally advanced disease. However, GnRH agonists have various adverse effects, such as increased fat mass and decreased insulin sensitivity in PCa patients, which may increase the risk of diabetes and cardiovascular disease.^[23]^ Previous research has confirmed the biological mechanisms linking MetS and PCa, although the specific mechanisms are not yet fully understood, and epidemiological studies have been contradictory.^[10]^ A study conducted abroad demonstrated that MetS and its components, particularly low high-density lipoprotein cholesterol levels and central obesity, were associated with an increased incidence of PCa. Prevention of MetS, the maintenance of high-density lipoprotein cholesterol levels, and the maintenance of a low waist circumference may be useful in reducing the incidence of PCa.^[27]^ However, a large-scale study in China, involving 482,943 consecutive males from 2010 to 2017, showed that MetS, hypertension, hyperlipidemia, hyperglycemia, and obesity were not associated with an increased risk of PCa. Advanced age and prostate-specific antigen levels were identified as risk factors for PCa.^[28]^ Another study in China indicated that PCa patients with concomitant MetS had higher levels of blood lipids and other metabolic indicators compared with those without MetS, suggesting that MetS may promote the development of PCa.^[29]^ More research is still needed to further explore this relationship in the future.

Keywords reflect the core themes and hot topics of an article, while burst keywords can predict future research trends and disciplinary frontiers. Key keywords related to the research field of PCa and MetS include metabolic syndrome, prostate cancer, risk, insulin resistance, men, and androgen deprivation therapy. Insulin resistance is a key pathogenic component of many metabolic diseases and is defined as a state of decreased responsiveness of insulin target tissues to physiological levels of insulin.^[30]^ It is currently unclear whether insulin resistance is a direct result of PCa treatment or is related to preexisting metabolic characteristics. Related studies suggest that future research needs to independently evaluate changes in glucose metabolism during PCa diagnosis and long-term hormone deprivation therapy, as this is of significant importance in distinguishing their unique contributions to the development of metabolic disorders.^[31]^ A recent study indicated that insulin resistance markers were not associated with the risk of clinically relevant PCa, but higher levels of glucose and the triglyceride-glucose (TyG) index were associated with poorer PCa survival.^[32]^ The TyG index is a marker of insulin resistance, and research by Li et al^[33]^ suggests that the TyG index may be a more accurate and effective predictor of PCa. Glucose metabolism may have a direct role in the progression of PCa, as leptin can stimulate angiogenesis and proliferation of PCa cells.^[34]^ Furthermore, insulin resistance involves elevated levels of insulin-like growth factor-1 and chronic inflammation, which may also promote the progression of PCa.^[32]^ Analysis of burst keywords shows that NF-kappa B, in vitro, receptor, meta-analysis, diagnosis, expression, and cholesterol have been the hot topics and trends in the research field over the past 5 years. Currently, researchers tend to conduct extensive studies to elucidate the molecular basis of disease progression, among which dysregulation of NF-kappa B activity has been considered a potential target for disease treatment, as it can lead to inflammatory diseases and cancer.^[35]^ Research has confirmed that NF-kappa B appears to play a key role in cell survival, proliferation, and invasion and can maintain the heterogeneity and multifocality of PCa.^[36]^ Additionally, NF-kappa B is a transcription factor that mediates the inflammatory response in MetS.^[37]^ Since 2021, cholesterol research has grown rapidly. Related research suggests that reducing dietary fat and cholesterol intake can slow the progression from latent lesions to PCa.^[38]^

However, this study also has certain limitations. First, although the literature in the Web of Science Core Collection database represents, to some extent, the highest level of research in this field internationally, other-language literature databases also have important academic status. This study did not include all the literature in this field, which may result in some bias in the results. Furthermore, some recently published high-quality articles may have been overlooked due to their short publication time and low citation frequency, resulting in a mismatch between the results of the bibliometric analysis and the actual situation. Finally, this study included all the literature related to the relationship between PCa and MetS that was retrieved, which may contain some literature with weak relevance to the research topic, but the impact of this literature on the overall results is relatively small.

## 5. Conclusion

This study conducted a visualization analysis of research related to PCa and MetS using the CiteSpace software, based on the Web of Science Core Collection database. It provided a comprehensive overview of this field from aspects such as publication quantity, authors, countries, journals, research institutions, and keywords, summarizing the current research hotspots and development trends in this area. Current research indicates the existence of biological principles linking PCa and MetS, but there is some controversy in epidemiology. Future studies still require large-scale prospective trials to further analyze the correlation between the 2, which has significant implications for guiding the prevention of PCa from the perspective of MetS.

## Author contributions

**Conceptualization:** Juan Wu, Wanjiao Wang.

**Supervision:** Qinghong Xi, Min Hu.

**Writing – original draft:** Feng Wang.
